# Low-frequency electrical stimulation alleviates immobilization-evoked disuse muscle atrophy by repressing autophagy in skeletal muscle of rabbits

**DOI:** 10.1186/s12891-022-05350-5

**Published:** 2022-04-28

**Authors:** A-Ying Liu, Quan-Bing Zhang, Hua-Long Zhu, Yong-Wei Xiong, Feng Wang, Peng-Peng Huang, Qi-Yu Xu, Hua-Zhang Zhong, Hua Wang, Yun Zhou

**Affiliations:** 1 Department of Rehabilitation Medicine, The Second Hospital of Anhui Medical University, No.678 Furong Road, Economic and Technological Development Zone, Hefei, 230601 China; 2 Key Laboratory of Environmental Toxicology of Anhui Higher Education Institutes, Hefei, China; 3 Department of Toxicology, School of Public Health, Anhui Medical University, Hefei, 230032 China; 4 Department of Orthopedics, The Second Hospital of Anhui Medical University, Hefei, China

**Keywords:** Disuse muscle atrophy, Low-frequency electrical stimulation, Autophagy, Knee extension contracture

## Abstract

**Background:**

The study aimed to investigate the effect of low-frequency electrical stimulation (LFES) on disuse muscle atrophy and its mechanism in a rabbit model of knee extension contracture.

**Methods:**

This study involved two experiments. In the time-point experiment, 24 rabbits were randomly divided into 4 groups: Control 1 (Ctrl1 group), immobilization for 2 weeks (I-2 group), immobilization for 4 weeks (I-4 group), and immobilization for 6 weeks (I-6 group). In the intervention experiment, 24 rabbits were randomly divided into 4 groups: Control 2 (Ctrl2 group), electrical stimulation (ESG group), natural recovery (NRG group), and electrical stimulation treatment (ESTG group). All intervention effects were assessed by evaluating the knee joint range of motion (ROM), cross-sectional area (CSA) of the rectus femoris muscle, and expression of autophagy-related proteins.

**Results:**

The time-point experiment showed that immobilization reduced the knee ROM, reduced the rectus femoris muscle CSA, and activated autophagy in skeletal muscle. The levels of five autophagy-related proteins [mammalian target of rapamycin (mTOR), phosphorylated mTOR (p-mTOR), autophagy-related protein 7 (Atg7), p62, and microtubule-associated protein light chain 3B-II (LC3B-II)] were significantly elevated in the skeletal muscle of the I-4 group. The intervention experiment further showed that LFES significantly improved the immobilization-induced reductions in ROM and CSA. Additionally, LFES resulted in a significant decrease in the protein expression of mTOR, p-mTOR, Atg7, p62, and LC3B-II in the rectus femoris muscle.

**Conclusions:**

LFES alleviates immobilization-evoked disuse muscle atrophy possibly by inhibiting autophagy in the skeletal muscle of rabbits.

**Supplementary Information:**

The online version contains supplementary material available at 10.1186/s12891-022-05350-5.

## Background

Trauma, joint fixation, nervous system damage, and prolonged bed rest may cause joint contracture, and long-term joint immobilization is a key factor in the development of joint contracture [1–4]. The knee is the largest and most complex joint of the human body, and it is the most important joint of the lower limbs with respect to walking and weight-bearing. The knee joint is easily injured by severe trauma, imbalanced weight-bearing, improper activity, and excessive loads [5]. Injured knee joints often require fixation [6], but prolonged inactivity can cause skeletal muscle atrophy and weakness. Studies have shown that the etiology of joint contracture can be divided into a myogenic component and articular component. In the early stage of joint contracture, myogenic factors are reversible in the natural recovery process, and progression of joint contracture to the stable stage is mainly caused by the irreversible articular component [7]. Myogenic contracture mainly manifests as disuse muscle atrophy caused by immobilization, and the cross-sectional area (CSA) of skeletal muscle fibers is significantly reduced [8–10]. Disuse muscle atrophy is considered to be an important part of myogenic contracture, which plays a role in promoting the occurrence and development of joint contracture [3]. The progression of skeletal muscle atrophy reduces physical activity, leading to a bedridden state. Therefore, preventing disuse muscle atrophy is very important to improve patients’ quality of life.

Our previous studies have shown that early treatment of disuse muscle atrophy is beneficial for rehabilitation of knee joint contracture [11]. Disuse muscle atrophy occurs secondary to accelerated proteolysis or decreased synthesis, and proteolysis plays a leading role in certain types of atrophy caused by inactivity [12]. Although all major proteolytic systems are involved in immobilization-triggered proteolysis in skeletal muscle, protein degradation induced by the autophagy–lysosomal pathway plays a key role in muscle atrophy [13, 14]. Speacht et al. [15] found that suspending the hind limbs of mice and fixing them with casts aggravated muscle atrophy by stimulating autophagy. In human skeletal muscle, the messenger RNA expression of five autophagy-related genes (*p62*, *LC3B*, *BECLIN-1*, *ATG12*, and *BNIP3*) increased during DonJoy splint fixation and returned to the baseline levels during rehabilitation training [16]. Correspondingly, studies have shown that muscle size can be maintained by repressing autophagy [17, 18]. Blocking the autophagy pathway with small interfering RNA or chloroquine can inhibit transforming growth factor β1-mediated skeletal muscle atrophy [19]. However, in the field of rehabilitation medicine, whether physical factor therapy can reduce disuse muscle atrophy by inhibiting autophagy remains unclear.

Electrical stimulation (ES) is a safe and effective physical factor therapy [20]. Under conditions of disuse, illness, and trauma, ES can enhance the contractile function of muscle fibers and prevent skeletal muscle atrophy [21, 22]. Low-frequency ES (LFES) was used to treat disuse skeletal muscle atrophy caused by tetrodotoxin paralysis in rats, and the results showed that LFES of two pulses per second was more effective than high-frequency ES [23]. In a model of chronic kidney disease-induced skeletal muscle atrophy, LFES improved protein metabolism and promoted skeletal muscle regeneration by up-regulating the insulin-like growth factor 1 signaling pathway [24]. LFES can improve disuse skeletal muscle atrophy, but no report has described the use of LFES to treat disuse muscle atrophy in joint contracture models. We hypothesized that LFES may improve disuse muscle atrophy by inhibiting immobilization-induced skeletal muscle autophagy.

In this study, rabbits were used to establish a clinical model of common knee extension contracture [25]. We first examined whether plaster external fixation of the lower limb could induce autophagy in rabbit skeletal muscle in a time-dependent manner. We then designed an intervention experiment to explore the role and mechanism of skeletal muscle autophagy in the improvement of disuse muscle atrophy by LFES.

## Methods

### Animals and experimental materials

Our procedures on rabbits were performed in accordance with the guidelines for humane treatment established by the Anhui Medical University (LLSC20190761). Forty-eight male skeletally mature New Zealand white rabbits (age, 3–4 months; weight, 2–2.5 kg) were purchased from the Experimental Animal Center of Anhui Medical University. The rabbits were individually reared in a cage of 60 × 50 × 40 cm^3^ at an ambient temperature of 24 °C and a 12−/12-h light/dark cycle. The rabbits had unlimited activity in the cage and were provided adequate food and water. All rabbits were fed a standard rabbit diet for 2 weeks before the experiment.

A Hwato SDZ-IV Electronic Acupuncture Treatment Instrument (Suzhou Medical Supplies Co., Ltd., Suzhou, China) was used in this study. The joint range of motion (ROM) measuring instrument (ZL201720251124.6) with utility model patent was designed by our research group, and the test–retest reliability value of the instrument was 0.826. Antibodies to autophagy-related protein 7 (Atg7) (ab133528), microtubule-associated protein light chain 3B-I/II (LC3B-I/II) (ab243506), and p62 (ab56416) were purchased from Abcam (Cambridge, UK). Mammalian target of rapamycin (mTOR) (2983S) and phosphorylated mTOR (p-mTOR) (5536S) antibodies were purchased from Cell Signaling Technology (Danvers, MA, USA). Glyceraldehyde 3-phosphate dehydrogenase (GAPDH) (F2612) antibody was purchased from Santa Cruz Biotechnology (Dallas, TX, USA). Thiazolyl blue tetrazolium bromide (MTT, M8180) was purchased from Solarbio (Beijing, China). A chemiluminescence detection kit was purchased from Thermo Fisher Scientific (Waltham, MA, USA).

### Grouping and intervention measures

The whole experiment was divided into two parts. The first part of the experiment was performed to explore the effects of immobilization on skeletal muscle autophagy, disuse muscle atrophy, and joint contracture. Twenty-four rabbits were randomly divided into four groups of six animals each: control 1 (Ctrl1 group), immobilization for 2 weeks (I-2 group), immobilization for 4 weeks (I-4 group), and immobilization for 6 weeks (I-6 group). The three groups of rabbits that were immobilized were anesthetized by injection of 30 mg/kg sodium pentobarbital through the ear vein, and the left knee joint was fixed in extension. In the Ctrl1 group, the rabbits moved freely for 6 weeks. In the I-2, I-4, and I-6 groups, plaster casts were used to immobilize the knee joint from the groin to the proximal interphalangeal joint at full extension [25], and the tubular plaster was removed at the end of each fixation time.

The second part of the experiment was performed to study the therapeutic effect and mechanism of LFES on disuse muscle atrophy and joint function. Twenty-four rabbits were randomly divided into four groups of six rabbits each: control 2 (Ctrl2 group), group of natural recovery after immobilization (NRG group), group of electrical stimulation treatment after immobilization (ESTG group), and pure ES (ESG group). The group characteristics are shown in Supplementary Material. In the Ctrl2 group, the rabbits were free to move for 7 weeks. In the ESG group, the rabbits were free to move for 4 weeks, followed by 10-Hz LFES treatment at 20 min per day for 3 weeks. In the NRG group, the rabbits’ left knee joint was fixed as described above, the plaster was removed after 4 weeks of immobilization, and natural recovery was allowed for 3 weeks. In the ESTG group, the left knee joint was fixed for 4 weeks and then the plaster was removed, and 10-Hz LFES was then performed at 20 min per day for 3 weeks.

### LFES treatment

Each rabbit in the ESG group and ESTG group received 3 weeks of 10-Hz LFES for 20 min once a day with a Hwato SDZ-IV Electronic Acupuncture Treatment Instrument. The intervention site for ESG was the quadriceps femoris of the left hind limb. First, the hair of the left hind leg was shaved off, and two 3- × 3-cm^2^ non-woven silica gel electrode sheets were then attached to the skin on the front side of the left hind leg. The distance between the two electrodes was 0.5 cm. The output current of the electronic acupuncture instrument was < 10 mA. We adjusted the size of the output current to cause quadriceps muscle contraction without strong resistance from the rabbit. A current of 5 mA caused obvious muscle contraction without excessive struggling by the rabbit in our preliminary experiment, so the current was set to 5 mA in our former experiment. The electronic acupuncture instrument was used in an intermittent wave mode with a pulse duration of 15 s and a pause time of 5 s.

### Tissue preparation and joint ROM measurement

Each rabbit’s left hind limb was dislocated at the left hip joint after euthanasia with an overdose of sodium pentobarbital via an auricular vein. This method of euthanasia was approved by the Animal Ethics Committee of Anhui Medical University (LLSC20190761). The starting point of the thigh muscles at the hip joint was cut off, and the left hind limb was completely detached from the torso. As in a previous experiment, a joint ROM measuring instrument was used to measure the ROM of the left knee joint [25]. The proximal end of the femur and distal ends of the tibia were fixed on the arthrometer with a metal clamp. All knee joints started at 0° of flexion before force was applied. The driving wheel was rotated to drive the dial to rotate; the tibia rotated indirectly while the femur remained motionless. Because the radius of the dial was fixed, the torque applied could be calculated by multiplying the force by the dial’s constant radius. In our previous experiment, we measured the ROM of normal rabbits and found that torque of 0.077 Nm could pull the knee joint to about 140° of buckling. After this, although the torque continued to increase, the bending angle of the knee joint was difficult to increase. Therefore, we used 0.077 Nm as the standard torque to measure the knee joint ROM in the present study. The surveyors were blinded to the rabbits’ grouping information. The ROM measurements were performed by two surveyors and repeated three times for each rabbit. The surveyors kept their measurements concealed from each other, and the buckling angle of each rabbit’s knee was the average of six measurements (contracture angle is shown in Supplementary Material). The following formulas were used to calculate the degree of contracture:

$$\mathrm{Total}\ \mathrm{contracture}=\mathrm{ROM}\ \mathrm{before}\ \mathrm{myotomy}\ \left(\mathrm{of}\ \mathrm{the}\ \mathrm{control}\ \mathrm{knee}\right)-\mathrm{ROM}\ \mathrm{before}\ \mathrm{myotomy}\ \left(\mathrm{of}\ \mathrm{the}\ \mathrm{contracted}\ \mathrm{knee}\right)$$

$$\mathrm{Arthrogenic}\ \mathrm{contracture}=\mathrm{ROM}\ \mathrm{after}\ \mathrm{myotomy}\ \left(\mathrm{of}\ \mathrm{the}\ \mathrm{control}\ \mathrm{knee}\right)-\mathrm{ROM}\ \mathrm{after}\ \mathrm{myotomy}\ \left(\mathrm{of}\ \mathrm{the}\ \mathrm{contracted}\ \mathrm{knee}\right)$$

$$\mathrm{Myogenic}\ \mathrm{contracture}=\left[\mathrm{ROM}\ \mathrm{before}\ \mathrm{myotomy}\ \left(\mathrm{of}\ \mathrm{the}\ \mathrm{control}\ \mathrm{knee}\right)-\mathrm{ROM}\ \mathrm{before}\ \mathrm{myotomy}\ \left(\mathrm{of}\ \mathrm{the}\ \mathrm{contracted}\ \mathrm{knee}\right)\right]-\left[\mathrm{ROM}\ \mathrm{after}\ \mathrm{myotomy}\ \left(\mathrm{of}\ \mathrm{the}\ \mathrm{control}\ \mathrm{knee}\right)-\mathrm{ROM}\ \mathrm{after}\ \mathrm{myotomy}\ \left(\mathrm{of}\ \mathrm{the}\ \mathrm{contracted}\ \mathrm{knee}\right)\right]$$

Three muscle tissue specimens of about 1 × 1 × 0.5 cm^3^ were subsequently removed from the middle of the separated rectus femoris muscle. Two specimens were used for hematoxylin and eosin (H&E) and immunofluorescence staining, and the other specimen was stored in a refrigerator at − 80 °C for the detection of skeletal muscle autophagy proteins.

### H&E staining

The rabbit rectus femoris tissues were fixed with 4% paraformaldehyde and then embedded in paraffin. The rectus femoris sections were stained using H&E. At 400× magnification, the cross section of the rectus femoris muscle was photographed with a Nikon TE2000-U microscope (Nikon, Tokyo, Japan), and four fields were randomly selected for each H&E-stained section. Image-Pro Plus 6.0 software (Media Cybernetics, Rockville, MD, USA) was used to count the number of muscle fibers and the total area of muscle fibers in each field. The average muscle fiber area under each field was statistically analyzed with SPSS Version 23.0 (IBM Corp., Armonk, NY, USA). The total number of myofiber cells is shown in Supplementary Material.

### Western blotting

An approximately 70- to 80-mg sample of rectus femoris tissue was obtained from the rectus femoris specimen, and 600 μL of lysis buffer (100:1 ratio of radio immunoprecipitation assay preparation and phenylmethylsulfonyl fluoride) was added to each sample. The homogenized tissue sample was transferred to a 1.5-mL Eppendorf tube, which was then placed into a centrifuge (4 °C). The tube was centrifuged at 12,000×*g* for 15 min. After centrifugation, 300 μL of the clear liquid in the intermediate layer was absorbed to prepare for the subsequent protein quantification by the bicinchoninic acid method. The antibody diluent was 5% skimmed milk (5 g powdered milk plus 100 mL Tris-buffered saline + Tween). The loading volume of the sample was determined according to the expression intensity of the protein. For the reference protein with relatively stable expression (GAPDH), the loading volume of 8 μg was sufficient; however, for a target protein with relatively weak expression, such as LC3B, the loading volume usually needed to reach 75 μg. For other target proteins, the loading volume was 48 μg for Atg7, 8 μg for p62, and 24 μg for mTOR and p-mTOR. The total lysate was separated with 12.5% sodium dodecyl sulfate–polyacrylamide gel electrophoresis buffer and then transferred onto polyvinylidene fluoride membranes. The membranes were first sealed with milk for 1.5 h and then incubated with primary antibodies for 1 to 2 h. The primary antibodies used were mouse anti-GAPDH (dilution ratio, 1:3000), rabbit anti-p-mTOR (1:1000), rabbit anti-mTOR (1:1000), rabbit anti-Atg7 (1:500), mouse anti-LC3 (1:1000), and mouse anti-p62 (1:1000). GAPDH was applied as a loading control. After being washed, the membranes were incubated with mouse anti-rabbit IgG or goat anti-mouse IgG at 1:10,000 to 1:50,000 dilution for 90 min, and the secondary antibodies were conjugated to horseradish peroxidase. The enhanced chemiluminescence reagent was then used for development. The signal was detected with a multipurpose imaging system (TY2019043988; Bio-Rad Laboratories, Hercules, CA, USA).

### Immunofluorescence

Thin sections (10 μm) of rectus femoris were fixed for 1 h with 4% paraformaldehyde. Nonspecific binding sites in the slides were blocked using 10% normal goat serum. The slides were incubated for 2 h with LC3B (1:200) at 37 °C. The slides were incubated with Alexa Fluor 488 conjugated secondary antibody (711–545-152, Jackson ImmunoResearch Laboratories, West Grove, PA, USA) for 90 min after washing with phosphate-buffered saline. The sections were stained with DAPI (C1002, Beyotime) for 5 min. All sections were then mounted and observed using a fluorescence microscope (BX53F; Olympus, Tokyo, Japan) under a 400× magnification field. Four fields were randomly selected and photographed for each slice. The number of LC3B-positive points in each visual field was counted for statistical analysis. The mean number of green fluorescence points in the individual muscle fibers in each visual field was calculated.

### Statistical analysis

An a priori power analysis was performed using data from our preliminary results on the ROM of the knee at different fixation times. We chose the most conservative sample size needed to detect differences with an alpha level of 0.05 and 80% power. This a priori power analysis indicated that our estimated required sample size was six rabbits per group. Because the observed effect sizes were slightly smaller than the expected values, we performed a retrospective power analysis, which determined that the ROM results were at 73% power.

Next, the Shapiro–Wilk test of normality was performed followed by one-way analysis of variance. The quantified data are presented as mean ± standard deviation. All data were entered and analyzed in SPSS Version 23.0 (IBM Corp.). One-way analysis of variance was used to assess the mean differences among groups of rabbits with respect to the ROM; CSA of the skeletal muscle fibers; and expression of mTOR, p-mTOR, Atg7, LC3B-I/II, and p62 proteins associated with muscle autophagy. When analysis of variance revealed differences, Bonferroni’s test or Tamhane’s T2 test was used to assess multiple comparisons between groups. A *P* value of < 0.05 was considered statistically significant.

## Results

### Immobilization induced disuse muscle atrophy and joint contracture in rabbits

**Fig. 1:**
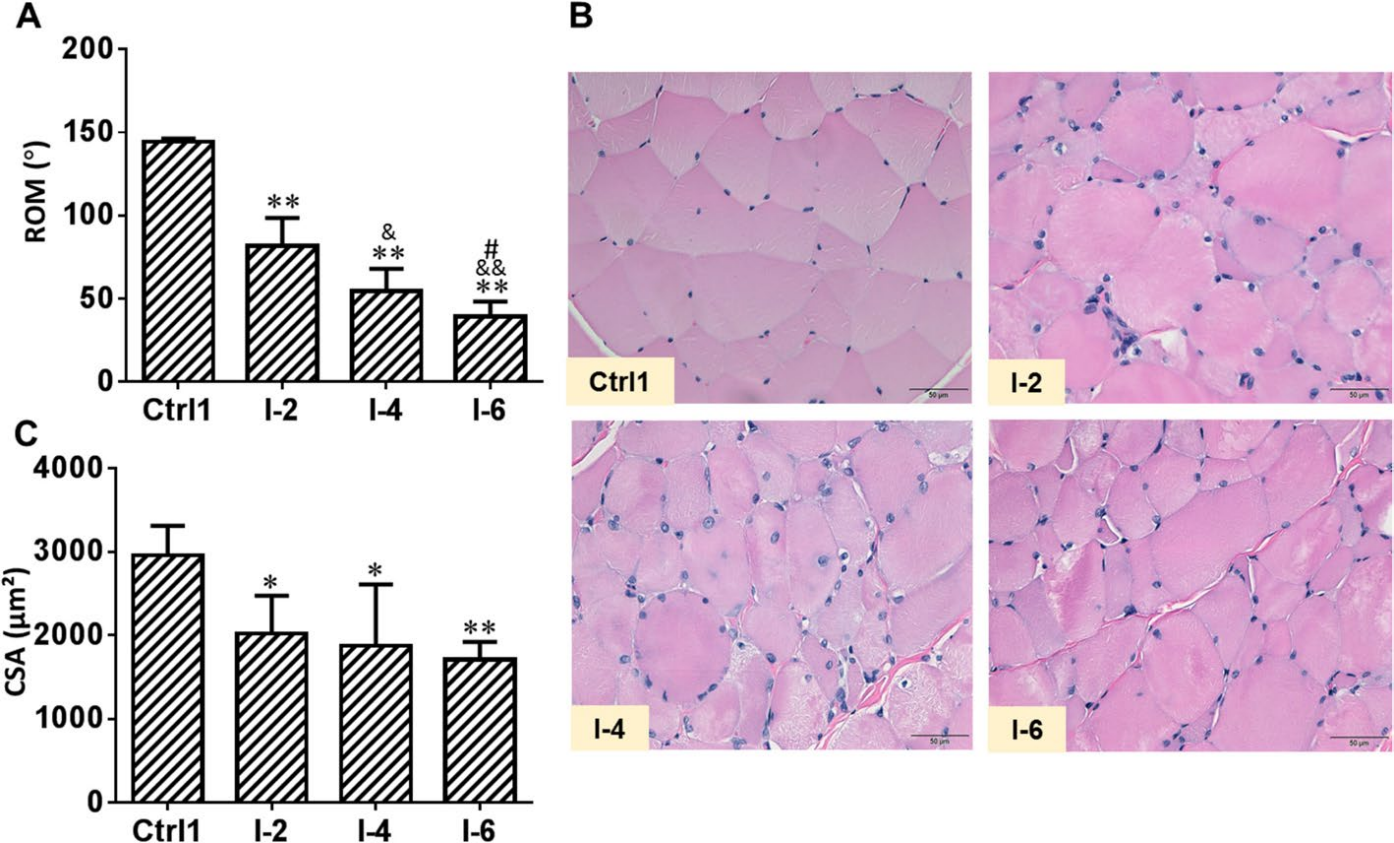
Immobilization induced disuse muscle atrophy and joint contracture in rabbits. The left hind limb of male rabbits was immobilized with a plaster bandage on the left knee joint in the extension position. The rectus femoris was dissected at 2, 4, and 6 weeks after immobilization. **A** Knee joint range of motion. **B** Representative images from the cross section of skeletal muscle fibers using hematoxylin and eosin staining. Scale bars represent 50 μm. **C** Quantitative analysis of cross-sectional area of skeletal muscle fibers. Ctrl1: control 1 group; I-2: immobilization for 2 weeks group; I-4: immobilization for 4 weeks group; I-6: immobilization for 6 weeks group. Data are expressed as mean ± SD. *n* = 6. ^*^P < 0.05, ^**^P < 0.01 compared with the Ctrl1 group; ^&^*P* < 0.05, ^&&^*P* < 0.01 compared with the I-2 group; ^#^*P* < 0.05 compared with the I-4 group

The knee joint flexion ROM in the four groups of rabbits is shown in Fig. 1A (Ctrl1 group: 144.27° ± 1.99°, I-2 group: 81.83° ± 16.64°, I-4 group: 54.48° ± 13.32°, I-6 group: 39.38° ± 8.83°). By comparing rabbits with different fixation times, we found that rabbits with longer fixation times had more significant reductions in knee ROM. After 2 weeks of immobilization, the knee flexion ROM was significantly lower in the I-2 group than in the Ctrl1 group (*P* < 0.01). The ROM in the I-4 group was further reduced compared with that in the I-2 group (*P* < 0.05). There was a statistically significant difference in the ROM between the I-4 group and Ctrl1 group (*P* < 0.01). The ROM of rabbits in the I-6 group was lower than that in the I-4 group (*P* < 0.05). There was a statistically significant difference in the ROM between the I-6 group and Ctrl1 group (*P* < 0.01). There was also a statistically significant difference in the ROM between the I-6 group and I-2 group (*P* < 0.01). As shown in Fig. 1B, the rectus femoris muscle disuse atrophy was more obvious in the groups with longer immobilization times. As shown in Fig. 1C, compared with the Ctrl1 group (CSA of 2962.89 ± 350.82 μm^2^), the I-6 group had the most obvious reduction in CSA (1711.81 ± 208.92 μm^2^) (*P* < 0.01). There was a statistically significant difference in the CSA between the I-2 group (2021.56 ± 451.15 μm^2^) and Ctrl1 group (*P* < 0.05) as well as between the I-4 group (1871.49 ± 737.48 μm^2^) and Ctrl1 group (*P* < 0.05). However, there was no significant difference in the CSA among the I-2, I-4, and I-6 groups (*P* > 0.05).

### Immobilization induced activation of rabbit skeletal muscle autophagy

**Fig. 2:**
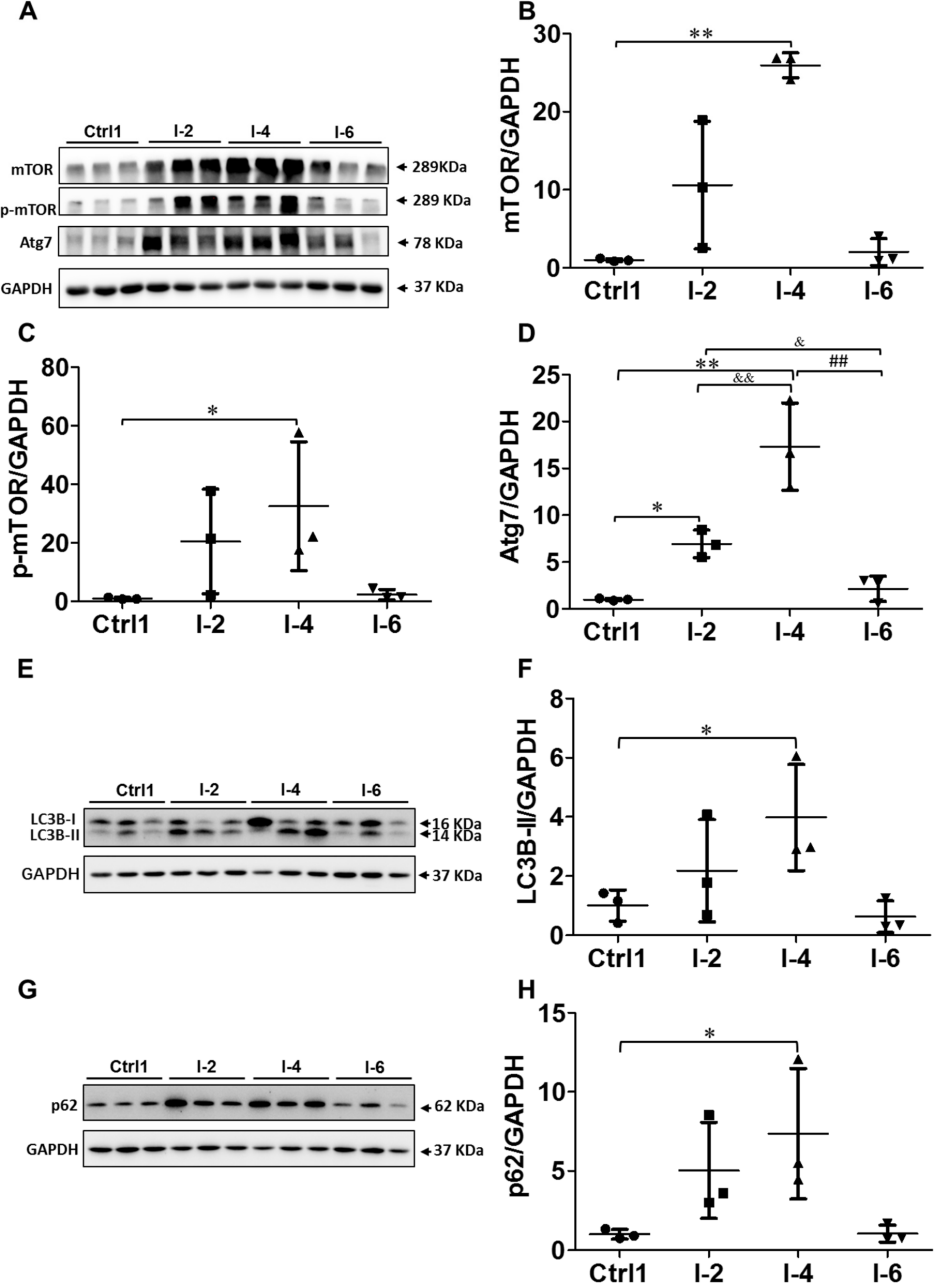
Immobilization induced activation of autophagy in rabbit skeletal muscle. The left hind limb of male rabbits was immobilized with a plaster bandage on the left knee joint in the extension position. The rectus femoris was dissected at 2, 4, and 6 weeks after immobilization. **A** Western blotting of mTOR, p-mTOR, and Atg7 proteins. **B** Quantitative analysis of mTOR. **C** Quantitative analysis of p-mTOR. **D** Quantitative analysis of Atg7. **E** Western blotting of LC3B-I/II protein. **F** Quantitative analysis of LC3B-II. **G** Western blotting of p62 protein. **H** Quantitative analysis of p62. Ctrl1: control 1 group; I-2: immobilization for 2 weeks; I-4: immobilization for 4 weeks; I-6: immobilization for 6 weeks; mTOR, mammalian target of rapamycin; p-mTOR, phosphorylated mammalian target of rapamycin; Atg7, autophagy-related protein 7; LC3B, microtubule-associated protein light chain 3B. Data are expressed as mean ± SD. *n* = 6. *P < 0.05, **P < 0.01 compared with the Ctrl1 group; ^&^*P* < 0.05, ^&&^*P* < 0.01 compared with the I-2 group; ^##^*P* < 0.01 compared with the I-4 group

The rectus femoris was used to test the effect of immobilization on autophagy in skeletal muscle. As shown in Fig. 2A–D, immobilization increased the expression levels of mTOR, p-mTOR, and Atg7 proteins in rabbit skeletal muscle. The protein expression level of mTOR in the I-4 group was higher than that in the Ctrl1 group (*P* < 0.01). The protein expression level of p-mTOR in the I-4 group was higher than that in the Ctrl1 group (*P* < 0.05). Atg7 expression was higher in the I-2 group than Ctrl1 group (*P* < 0.05) and higher in the I-4 group than in the Ctrl1 and I-2 groups (*P* < 0.01). Atg7 expression in the I-6 group was lower than that in the I-2 group (*P* < 0.05) and I-4 group (*P* < 0.01).

**Fig. 3:**
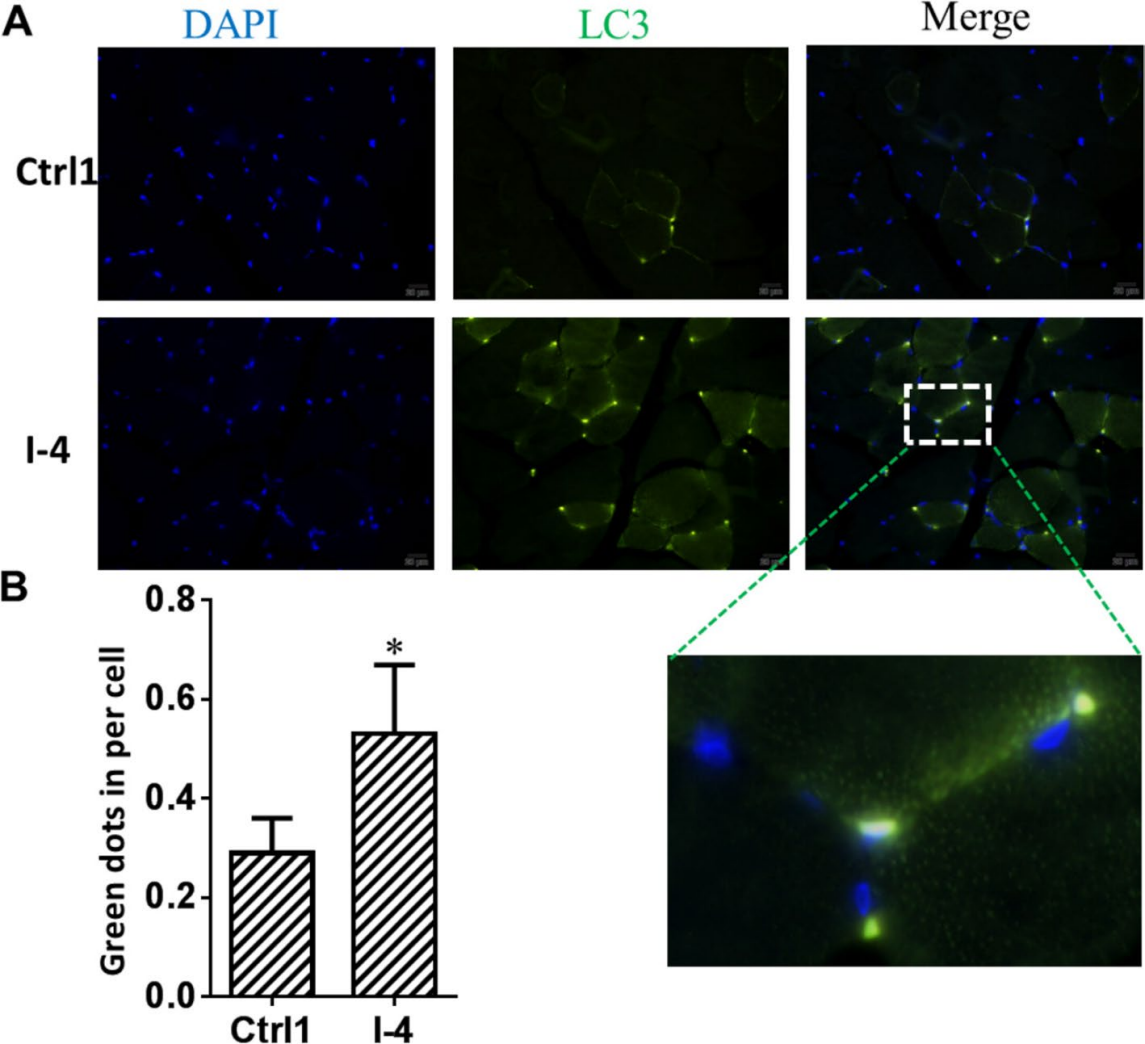
Immobilization increased LC3 puncta in rabbit skeletal muscle. The left hind limb of male rabbits was immobilized with a plaster bandage on the left knee joint in the extension position. The rectus femoris was dissected at 2, 4, and 6 weeks after immobilization. **A** Representative images from control 1 group and 4-week immobilization group using immunostaining for LC3B. Scale bars represent 20 μm. **B** Quantitative analysis of immunostaining of LC3B. Ctrl1: control 1 group; I-4: immobilization for 4 weeks; LC3B, microtubule-associated protein light chain 3B. Data are expressed as mean ± SD. *n* = 6. ^*^P < 0.05 compared with the Ctrl1 group

As shown in Fig. 2E–H, immobilization caused an increase in the LC3B-II and p62 protein levels in rabbit skeletal muscle. The protein expression levels of LC3B-II and p62 in the I-4 group were higher than those in the Ctrl1 group (*P* < 0.05). As shown in Fig. 3A and B, LC3 immunofluorescence staining of frozen sections of rectus femoris provided further evidence of autophagy in atrophic skeletal muscle. The results showed a significantly higher number of LC3-positive points in the I-4 group than in the Ctrl1 group (*P* < 0.05) (Fig. 3B).

### LFES improved disuse muscle atrophy and knee joint contracture

**Fig. 4:**
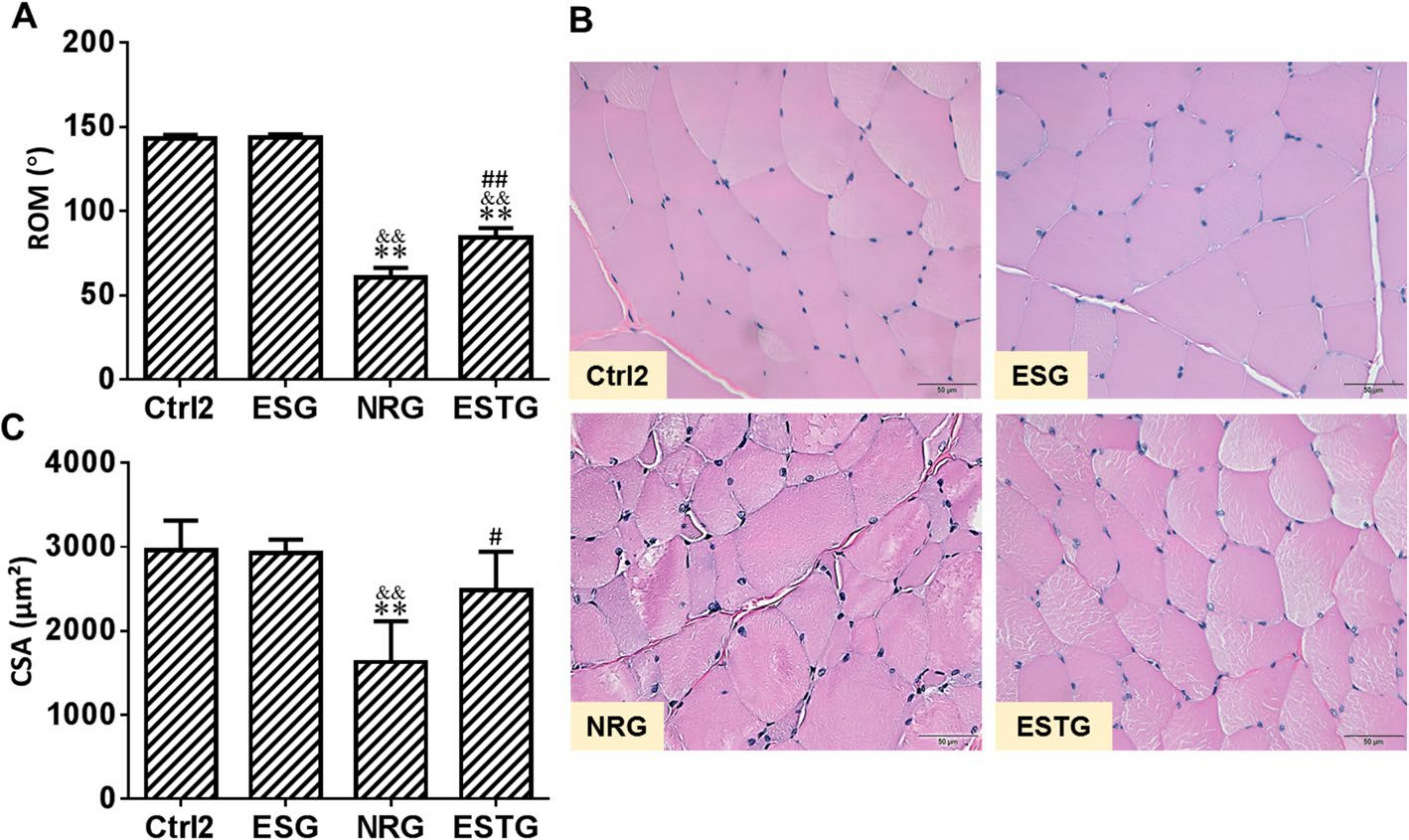
Low-frequency electrical stimulation (LFES) improved disuse muscle atrophy and knee joint contracture. Male rabbits were treated with LFES on the quadriceps femoris muscle for 3 weeks after 4 weeks of immobilization. The rectus femoris was dissected after 3 weeks of LFES. **A** Knee joint range of motion. **B** Representative images from the cross section of skeletal muscle fibers using hematoxylin and eosin staining. Scale bars represent 50 μm. **C** Quantitative analysis of cross-sectional area of skeletal muscle fibers. Ctrl2: control 2 group; ESG: electrical stimulation group; NRG: natural recovery group; ESTG: electrical stimulation treatment group. Data are expressed as mean ± SD. *n* = 6. ^**^P < 0.01 compared with the Ctrl2 group; ^&&^*P*<0.01 compared with the ESG group; ^#^*P*<0.05, ^##^*P*<0.01 compared with the NRG group

The effect of LFES on rabbit knee joint ROM is shown in Fig. 4A. Knee joint ROM in the NRG group (60.67° ± 5.71°) and ESTG group (84.27° ± 5.66°) was significantly lower than that in the Ctrl2 group (143.33° ± 2.14°) and ESG group (143.82° ± 1.93°) (*P* < 0.01). The improvement of ROM in the ESTG group was significantly greater than that in the NRG group (*P* < 0.01). As shown in Fig. 4B, the improvement of skeletal muscle disuse atrophy in the ESTG group was greater than that in the NRG group. The quantitative results in Fig. 4C show that the CSA in the NRG group (1628.99 ± 486.12 μm^2^) was significantly smaller than that in the Ctrl2 group (2962.89 ± 350.82 μm^2^) and ESG group (2928.44 ± 160.23 μm^2^) (*P* < 0.01), and the CSA in the ESTG group (2486.92 ± 455.99 μm^2^) was significantly improved compared with that in the NRG group (*P* < 0.05).

### LFES reversed immobilization-triggered activation of autophagy in rabbit rectus femoris

**Fig. 5:**
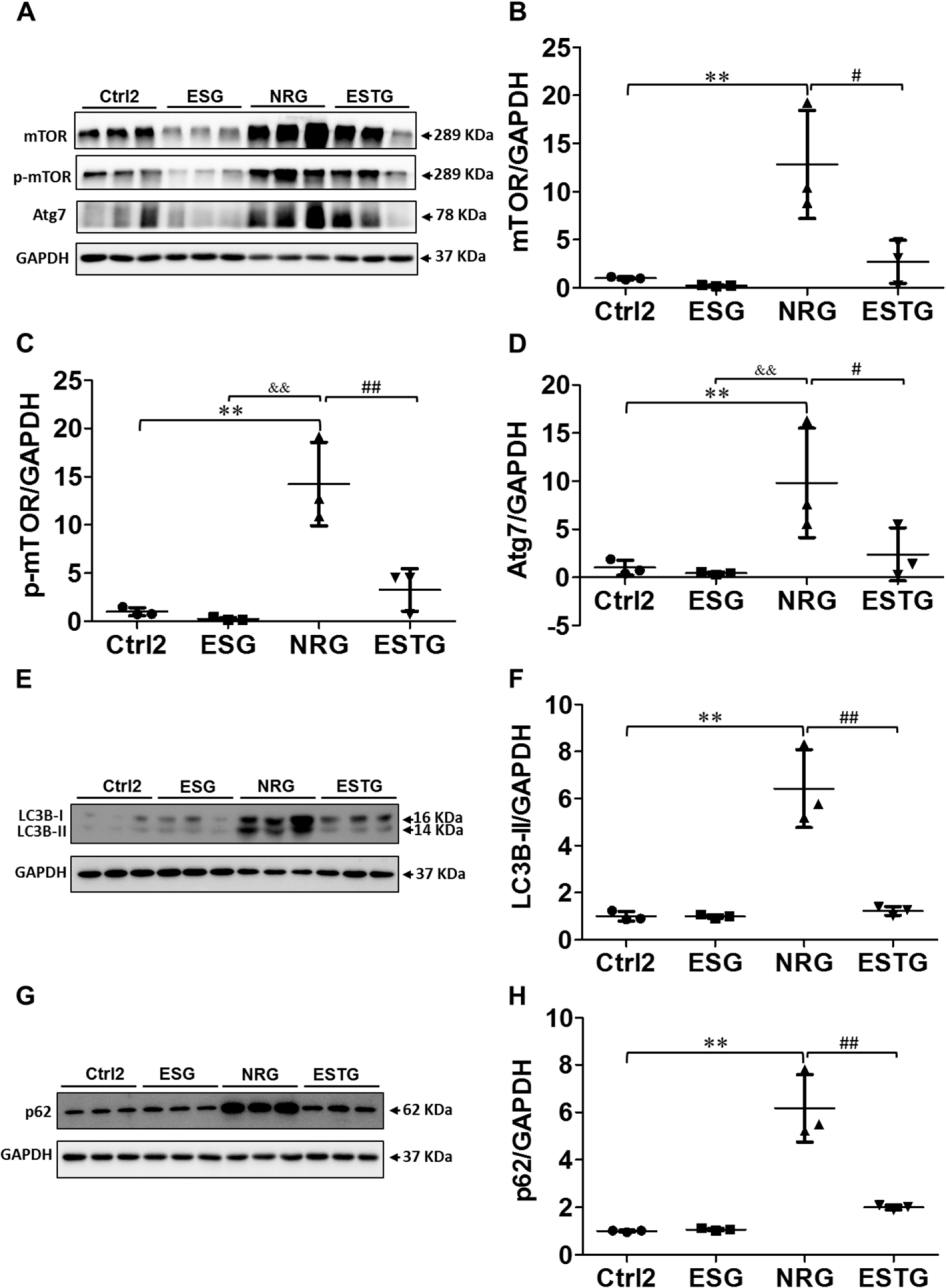
Low-frequency electrical stimulation (LFES) alleviated immobilization-triggered activation of autophagy in rabbit skeletal muscle. Male rabbits were treated with LFES on the quadriceps femoris muscle for 3 weeks after 4 weeks of immobilization. The rectus femoris was dissected after 3 weeks of LFES. **A** Western blotting of mTOR, p-mTOR, and Atg7 proteins. **B** Quantitative analysis of mTOR. **C** Quantitative analysis of p-mTOR. **D** Quantitative analysis of Atg7. **E** Western blotting of LC3B-I/II protein. **F** Quantitative analysis of LC3B-II. **G** Western blotting of p62 protein. **H** Quantitative analysis of p62. Ctrl2: control 2 group; ESG: electric stimulation group; NRG: natural recovery group; ESTG: electrical stimulation treatment group; mTOR, mammalian target of rapamycin; p-mTOR, phosphorylated mammalian target of rapamycin; Atg7, autophagy-related protein 7; LC3B, microtubule-associated protein light chain 3B. Data are expressed as mean ± SD. *n* = 6. ^**^P < 0.01 compared with the Ctrl2 group; ^&&^*P* < 0.01 compared with the ESG group; ^#^*P* < 0.05, ^##^*P* < 0.01 compared with the NRG group

The expression levels of mTOR, p-mTOR, and Atg7 proteins in rectus femoris were detected to examine the role of autophagy inhibition in the treatment of knee joint contracture by LFES. As shown in Fig. 5A–D, LFES inhibited the expression of mTOR, p-mTOR, and Atg7 proteins in rabbit skeletal muscle. The protein expression level of mTOR in the NRG group was higher than that in the Ctrl2 group (*P* < 0.01). The mTOR expression in the ESTG group was lower than that in the NRG group (*P* < 0.05). The protein expression level of p-mTOR in the NRG group was higher than that in the Ctrl2 and ESG groups (*P* < 0.01). The p-mTOR expression in the ESTG group was lower than that in the NRG group (*P* < 0.01). The protein expression level of Atg7 in the NRG group was higher than that in the Ctrl2 and ESG groups (*P* < 0.01). The Atg7 expression in the ESTG group was lower than that in the NRG group (*P* < 0.05).

The expression levels of LC3B-II and p62 proteins in rectus femoris were also detected to further explore the therapeutic effect of LFES on joint contracture. As shown in Fig. 5E–H, the expression levels of LC3B-II and p62 in the NRG group were significantly higher than those in the Ctrl2 group (*P* < 0.01). Compared with the NRG group, the expressions of LC3B-II and p62 in the ESTG group were significantly inhibited (*P* < 0.01).

## Discussion

The flexion-type knee joint contracture model has been used in most experimental studies of knee joint contracture to date [26, 27]. When modeling, the animal’s knee joint is fixed at about 150° of flexion, which leads to limited knee extension [4, 28–30]. According to the internationally accepted neutral-zero method, the neutral position of the knee joint is the extension position, which is defined as 0°. The ROM of a normal knee joint is about 120° to 150° in flexion and 5° to 10° in hyperextension [31]. The functional position of the knee joint is 20° to 30° of flexion [32]. Knee joint injuries usually require fixation in an extended or functional position to promote tissue healing. Therefore, fixation in the extension position or functional position is a common orthopedic treatment for knee trauma or other musculoskeletal diseases [33]. To ensure consistency with clinical practice, we used tubular plaster external fixation to establish a rabbit model of knee extension contracture [25]. Because of individual differences and difficulty in cooperation of rabbits, the reliability of knee joint ROM measurement in vivo is difficult to guarantee. Therefore, we separated the left hind limb of the rabbits to measure the ROM of the knee joint. Compared with in vivo measurements, this in vitro measurement method excludes the interference induced by the activity of live rabbits. The same fixed torque can be maintained during the measurement of different individuals [11, 25, 34]. As mentioned above, our model of extension knee contracture is more suitable for patients’ clinical situations than other models of flexion knee contracture.

Skeletal muscles play an important role in vital body functions such as breathing and movement [35–37]. One study showed that the skeletal muscle weight and CSA were significantly reduced in a model of suspension-induced disuse muscle atrophy [38]. Our time-point experiment showed the development of disuse atrophy of skeletal muscle during the formation of fixation-induced knee joint contracture in rabbits. Quantitative analysis by H&E staining showed that skeletal muscle atrophy progressed rapidly during the first 2 weeks of fixation and then progressed slowly from 2 to 6 weeks of fixation. Cessation of skeletal muscle use could lead to muscle atrophy, which is reversible after a short period of non-use [39]. Our intervention experiment showed that the indexes of CSA and ROM in the NRG group did not return to the normal level compared with those in the Ctrl2 group, indicating that short-term natural recovery after 4 weeks of fixation could not completely reverse skeletal muscle atrophy and joint mobility limitation. Compared with the NRG group, the ROM and CSA in the ESTG group were significantly improved. These results indicate that LFES has a certain therapeutic effect on improving joint function and disuse muscle atrophy.

Although all major proteolytic systems are involved in inactivity-induced proteolysis in skeletal muscle, there is increasing evidence that the autophagy–lysosomal pathway plays an important role [40, 41]. No studies to date have investigated the effect of the autophagolysosomal system on disuse muscle atrophy in a knee joint contracture model. In a rat model of denervation-induced disuse muscle atrophy, pre-lysosomal autophagy flux was upregulated at 1 and 3 days post-denervation but was reduced compared with the time-matched sham-operated controls at 7 days post-denervation [42]. In a mouse model of suspension-induced disuse muscle atrophy, skeletal muscle unloading resulted in increased mitophagy and decreased mitochondrial biogenesis regulation [43]. In the present study, we focused on the effect of skeletal muscle autophagy on early disuse muscle atrophy in a rabbit model of knee joint contracture. p-mTOR and Atg7 are important upstream regulators of autophagy [44, 45]. During the formation of autophagosomes, LC3-II specifically binds to autophagosomes or autophagolysosomes, and the increased expression of LC3-II is a signal of autophagy activation [46–48]. As one of the most important substrates of autophagy, p62 is a vital receptor in autophagy and is generally regarded as an indicator of autophagy degradation [49, 50]. Our results showed that immobilization significantly increased the overexpression of four autophagy-specific protein markers (p-mTOR, Atg7, LC3B-II, and p62) in rabbit skeletal muscle. As indicated by these markers, skeletal muscle autophagy reached the highest level after 4 weeks of fixation and then decreased after 6 weeks of fixation. The potential physiological mechanisms contributing to the decreased levels of autophagy regulators after 6 weeks of immobilization may involve increased lysosomal impairment and oxidative stress [42]. Although the autophagy of skeletal muscle was reduced in the I-6 group, the atrophy of skeletal muscle in the I-6 group was the most obvious. This result may have been related to the fixation time and the activation of other protein degradation pathways. In conclusion, plaster external fixation during the development of joint contracture triggers disuse atrophy of skeletal muscle by activation of the autophagy pathway mediated by p-mTOR signaling.

LFES has been shown to improve muscle atrophy to a certain extent [23, 51, 52]. Because the autophagy of skeletal muscle in the I-4 group increased to the greatest extent, we performed LFES on the quadriceps muscle after 4 weeks of fixation in the intervention experiment. Our results also showed that the expression of autophagy-related proteins in the ESTG group was significantly lower than that in the NRG group. The skeletal muscle CSA and knee ROM in the ESTG group were significantly improved compared with those in the NRG group.

This study has three major limitations: the small sample size (six rabbits per group), the inclusion of only male rabbits, and the short-term treatment period (only 3 weeks). Because of the limitations of the experimental conditions, we only used LFES to treat disuse muscle atrophy caused by knee joint immobilization. The results showed that disuse muscle atrophy and joint contracture caused by immobilization were not completely reversed. We anticipate that if LFES is used in combination with other rehabilitation methods and prolonged treatment, disuse muscular atrophy and knee mobility limitation during contracture could be significantly improved.

## Conclusion

Short-term exposure to immobilization induced disuse muscle atrophy and skeletal muscle autophagy in this rabbit model of extension knee joint contracture. LFES protected against immobilization-induced disuse muscle atrophy, which was associated with autophagy inhibition in the skeletal muscle of rabbits. These data provide an experimental basis for earlier prevention and treatment of disuse muscle atrophy caused by immobilization.

## Supplementary Information

**Additional file 1: Supplementary Material includes supplementary table S1** (Grouping features of part 2.), **supplementary table S2** (Total contracture, myogenic contracture, arthrogenic contracture and fiber number data.) and the original versions of gels and blots images.

## Data Availability

The datasets used and/or analyzed during the current study are available from the corresponding author on reasonable request.

## Abbreviations

Ctrl1: Control 1 group
I-2: Immobilization for 2 weeks group
I-4: Immobilization for 4 weeks group
I-6: Immobilization for 6 weeks group
Ctrl2: Control 2 group
ESG: Electrical stimulation group
NRG: Natural recovery group
ESTG: Electrical stimulation treatment group
ROM: Range of motion
CSA: Cross-sectional area
LFES: Low-frequency electrical stimulation
mTOR: Mammalian target of rapamycin
p-mTOR: Phosphorylated-mechanistic target of rapamycin
Atg7: Autophagy related gene 7
LC3: Microtubule-associated protein 1 light chain 3
p62: Sequestosome 1 protein
GAPDH: Glyceraldehyde phosphate dehydrogenase
