# Supplementary material for: Low-frequency electrical stimulation alleviates immobilization-evoked disuse muscle atrophy by repressing autophagy in skeletal muscle of rabbits
Source: BMC Musculoskelet Disord. 2022 Apr 28;23:398. doi: 10.1186/s12891-022-05350-5 (PMC9047266; doi:10.1186/s12891-022-05350-5)
Supplement: Supplementary file 1 — Additional file 1: Supplementary Material includes supplementary table S1 (Grouping features of part 2.), supplementary table S2 (Total contracture, myogenic contracture, arthrogenic contracture and fiber number data.) and the original versions of gels and blots images. [file 12891_2022_5350_MOESM1_ESM.doc]

**Supplementary table S1** Grouping features of part 2.

| **Groups** | **Number** | **Immobilization time(w)** | **Free cage time(w)** | **electrical stimulation** |
| --- | --- | --- | --- | --- |
| Ctrl2  ESG  NRG  ESTG | 6  6  6  6 | 0  0  4  4 | 7  7  3  3 | _ +  _  + |

Ctrl2: control 2 group; ES: electric stimulation group; NRG: natural recovery group; ESTG: electrical stimulation treatment group. Rabbits of natural recovery group (NRG) and electrical stimulation treatment group (ESTG) went through four weeks of fixation before three weeks of free cage activity.

**Supplementary table S2** Total contracture, myogenic contracture, arthrogenic contracture and fiber number data

| Group | Total  Contracture(°) | Myogenic Contracture(°) | Arthrogenic Contracture(°) | Fiber Number |
| --- | --- | --- | --- | --- |
| Ctrl1  I-2  I-4  I-6  Ctrl2  ESG  NRG  ESTG | 0.00±0.00  62.43±15.78^a^  89.78±12.26 ^ab^  104.88±15.72 ^abc^  0.00±0.00  0.00±0.00  74.07±1.91^de^  59.07±4.95^def^ | 0.00±0.00  35.67±10.80 ^a^  48.77±10.17 ^ab^  49.10±8.96 ^abc^  0.00±0.00  0.00±0.00  36.38±2.77^de^  24.50±1.40^def^ | 0.00±0.00  26.77±5.86 ^a^  41.02±3.39 ^ab^  55.78±2.49 ^ab^  0.00±0.00  0.00±0.00  37.68±2.46^de^  34.57±1.47^de^ | 29.44±3.79  42.61±4.39^a^  44.92±7.16^a^  47.88±1.83 ^a^  29.94±4.14  29.50±3.50  49.69±9.95^de^  33.38±6.47^f^ |

Data were expressed as *mean* ± *S.D*. *^a^P<*0.01 compared with Ctrl1; ^b^*P <*0.01 compared with I-2; ^c^*P <*0.01 compared with I-4; *^d^P<*0.01 compared with Ctrl2; ^e^*P <*0.01 compared with ES; ^f^*P <*0.01 compared with NR.

**The original, unprocessed versions of gels and blots images.** The images contain markers for molecular marker. There is one GAPDH band in Figure D did not find the image containing molecular markers due to the long end time of the experiment.

**A**


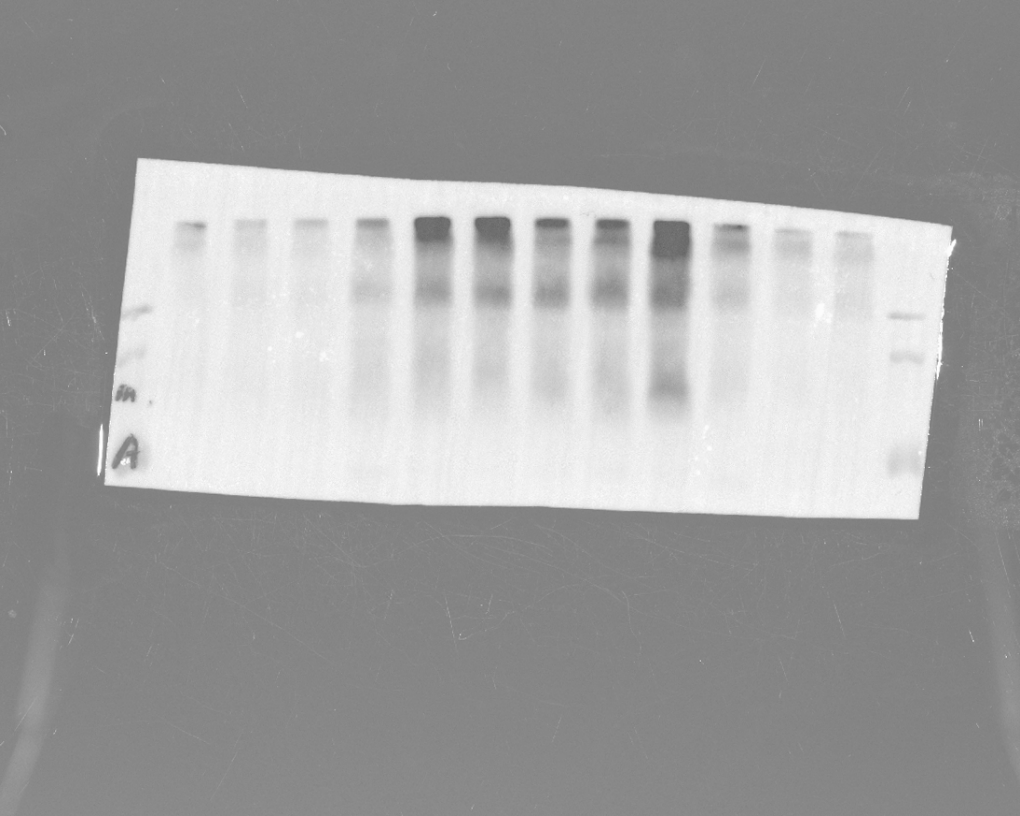

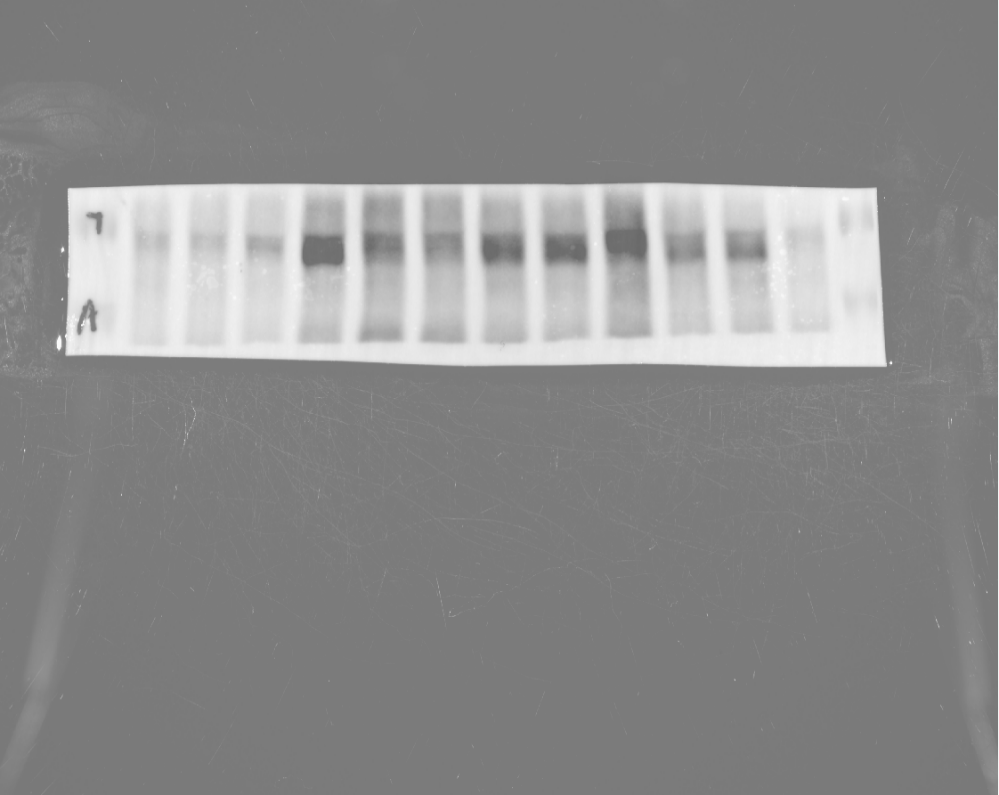

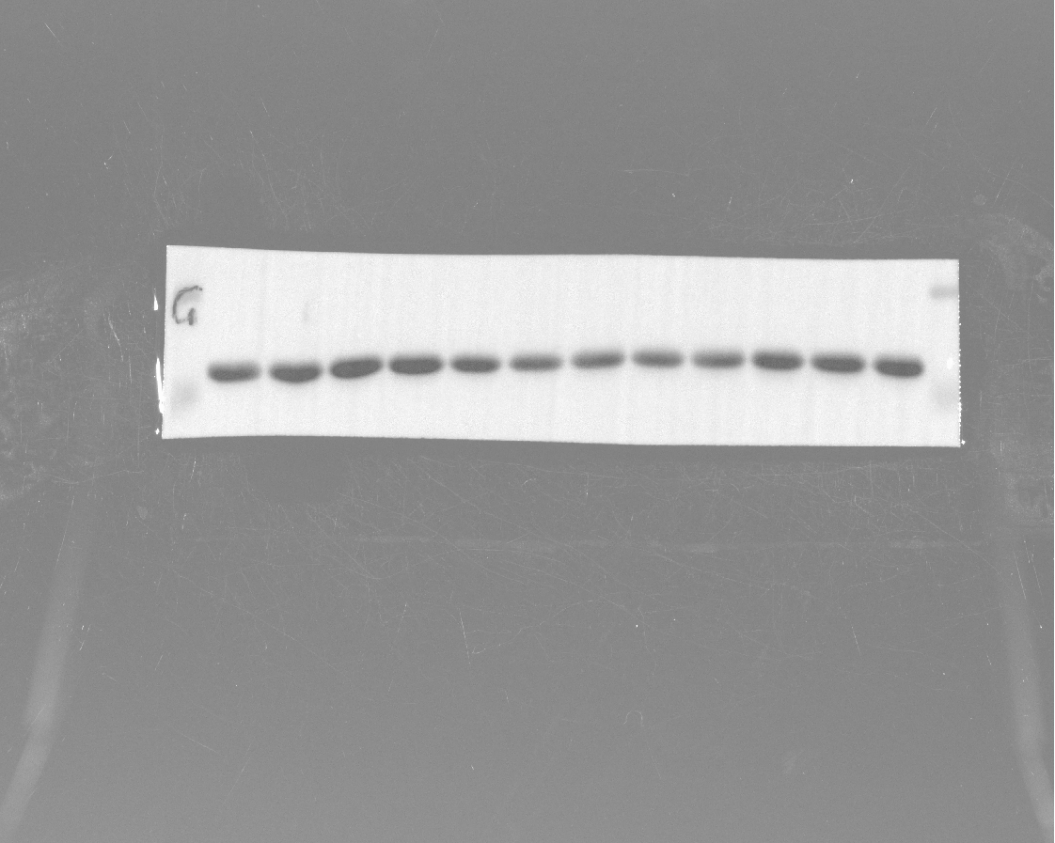


**p-mTOR**

**Atg7**

**GAPDH**

**34 KDa**

**72 KDa**

**180 KDa**

**180 KDa**

**Ctrl1**

**I-2**

**I-4**

**I-6**


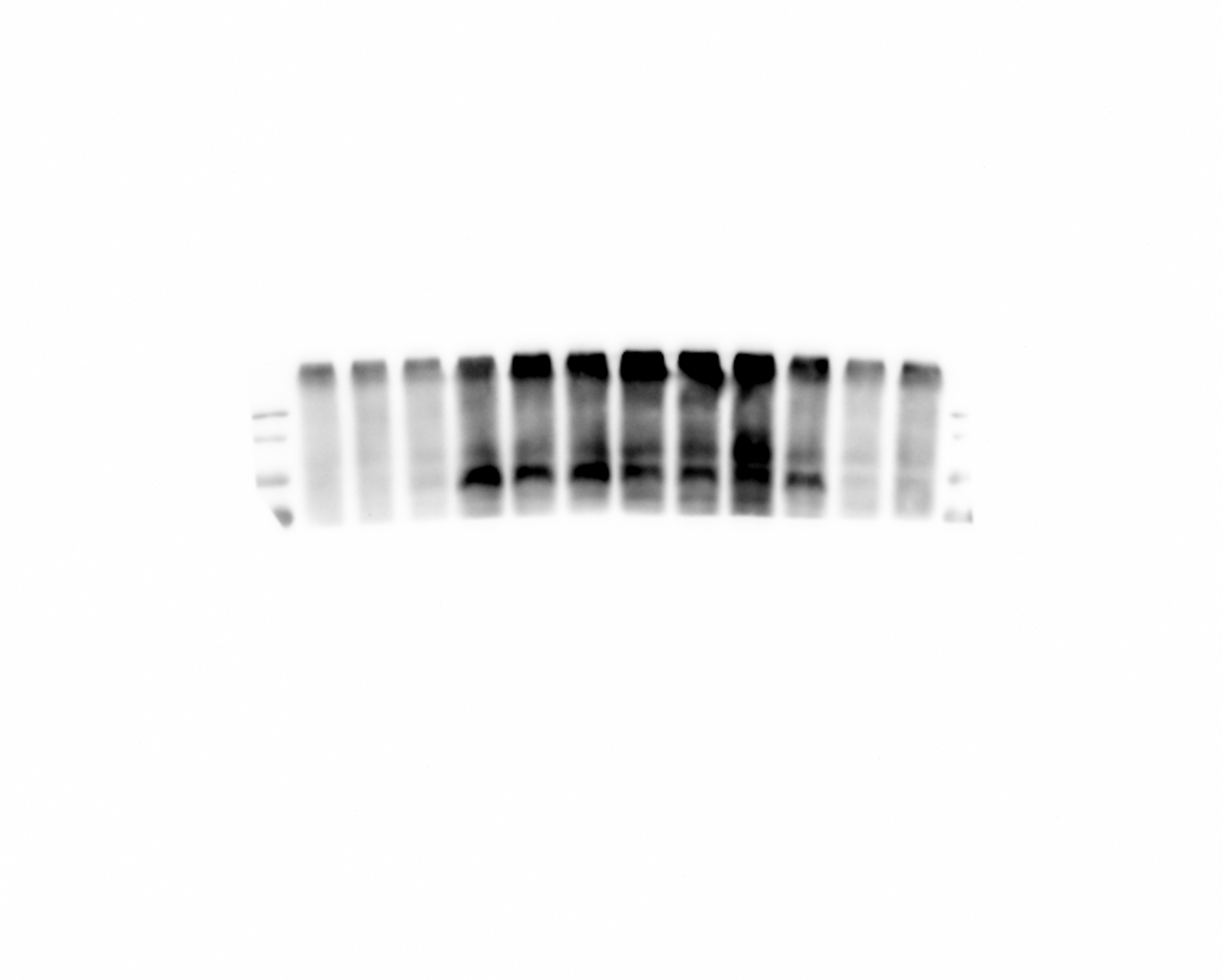


**mTOR**


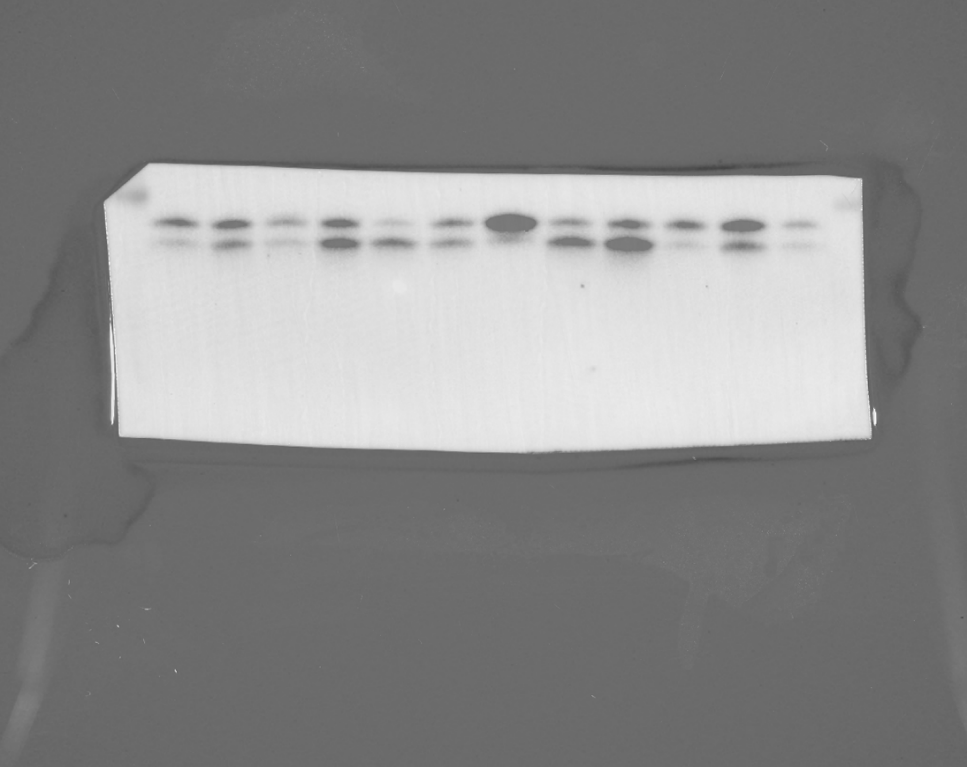

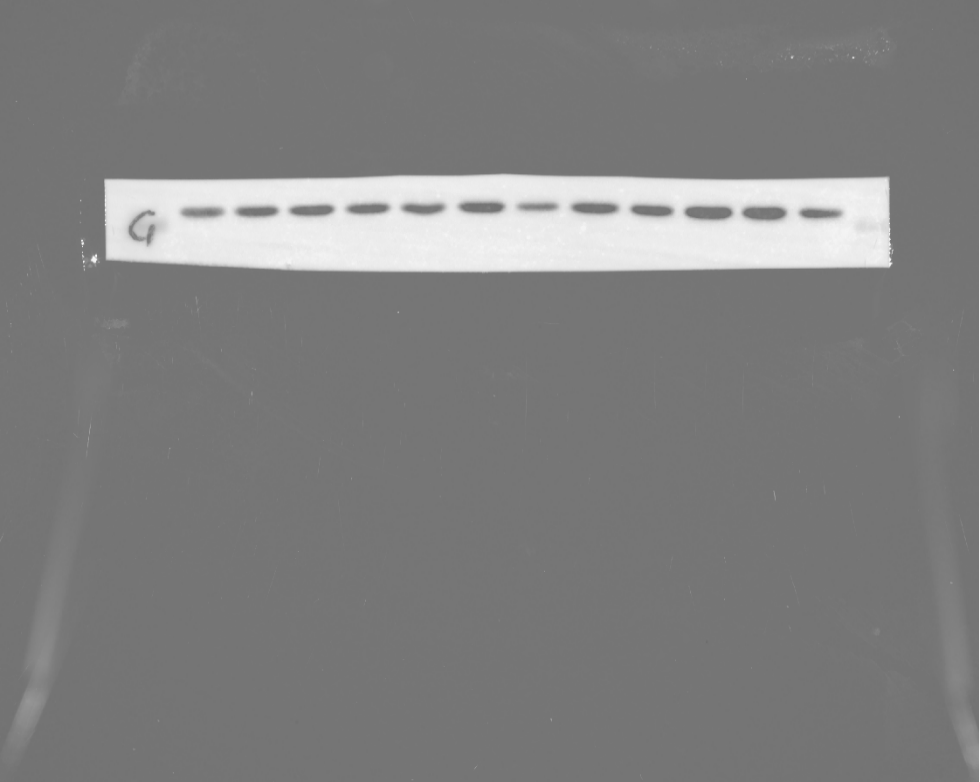


**LC3B-II**

**LC3B-I**

**17 KDa**

**34 KDa**


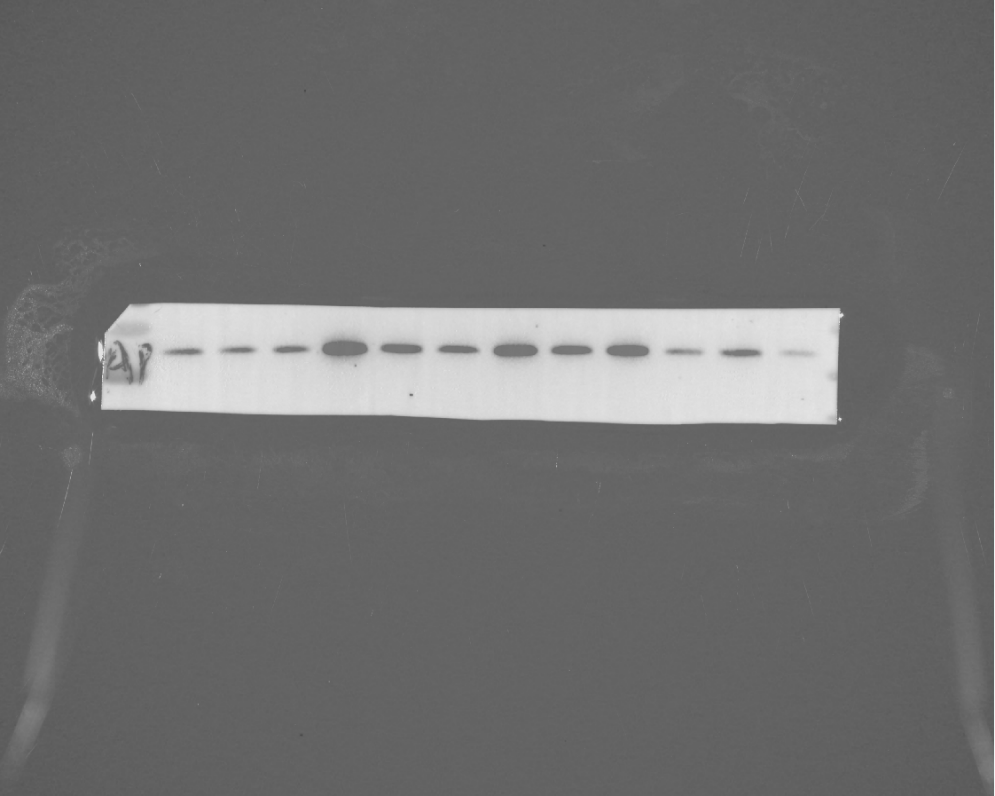


**GAPDH**

**p62**

**55KDa**


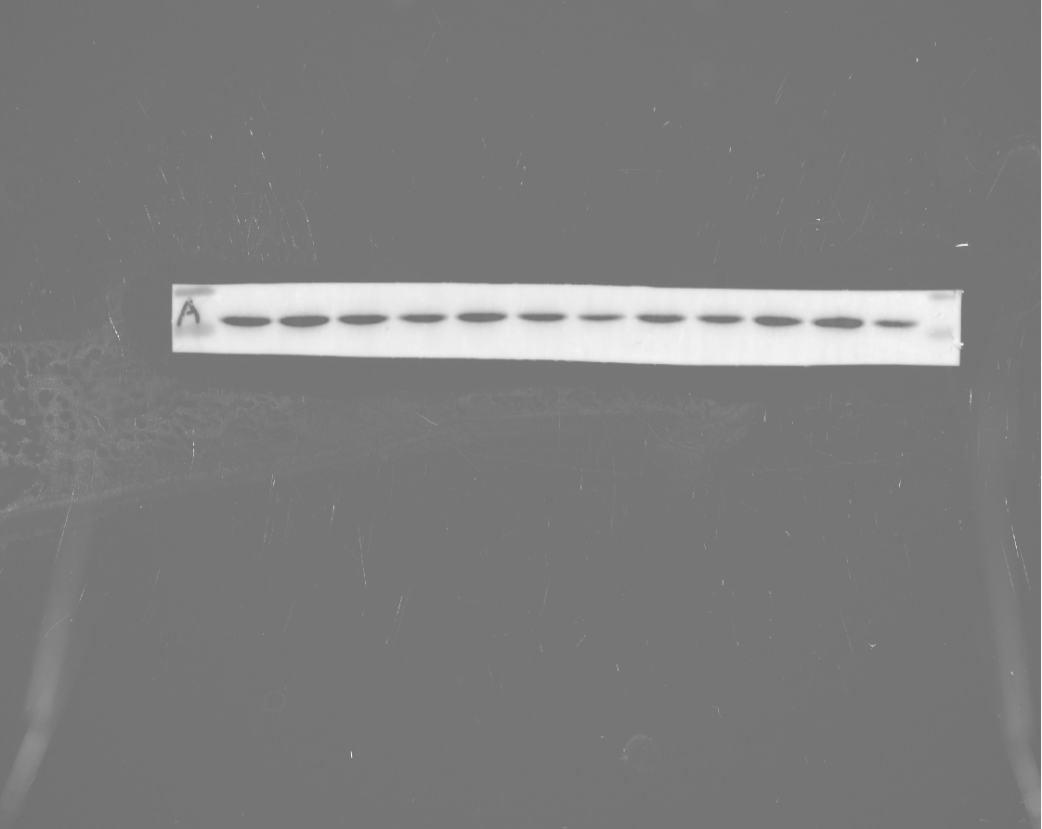


**GAPDH**

**34 KDa**

**Ctrl1**

**I-2**

**I-4**

**I-6**

**B**


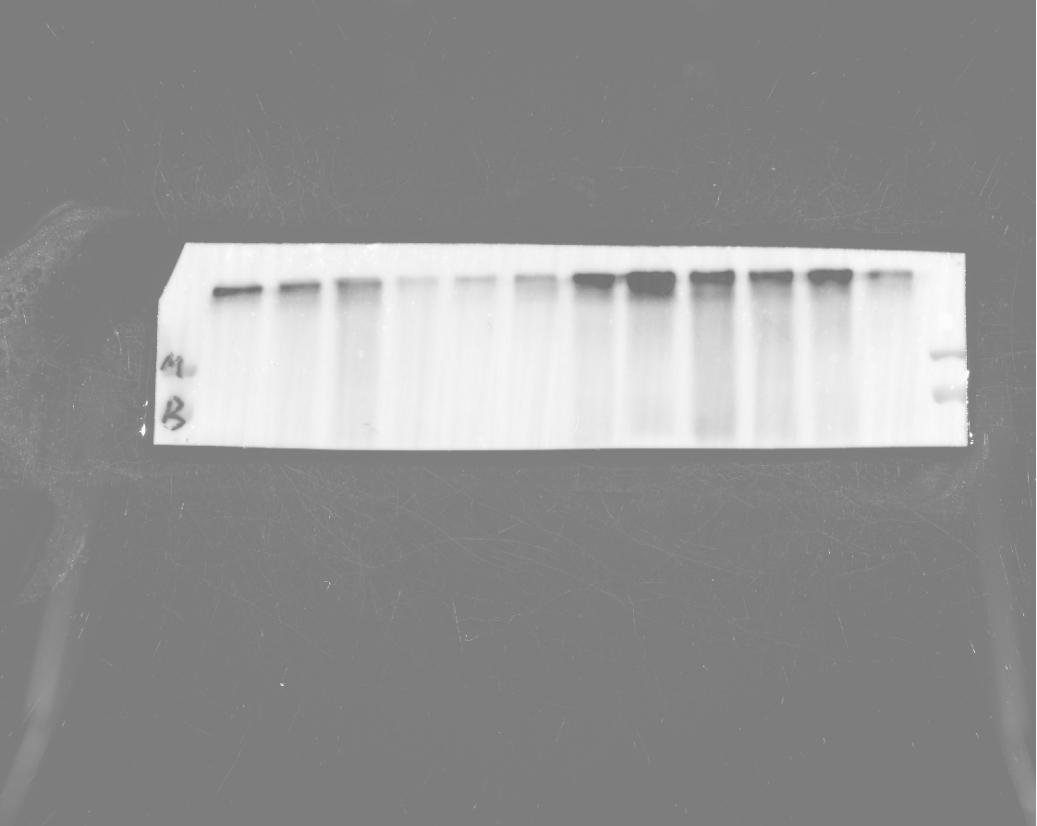

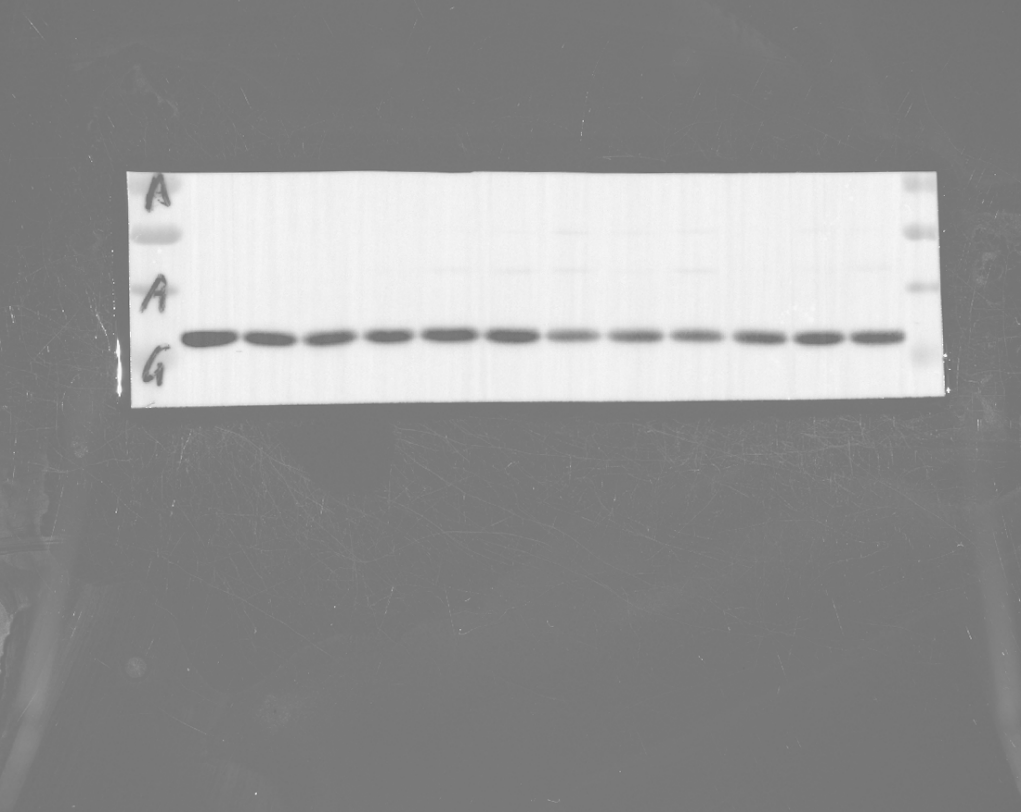


**180 KDa**

**180 KDa**

**95 KDa**

**34 KDa**


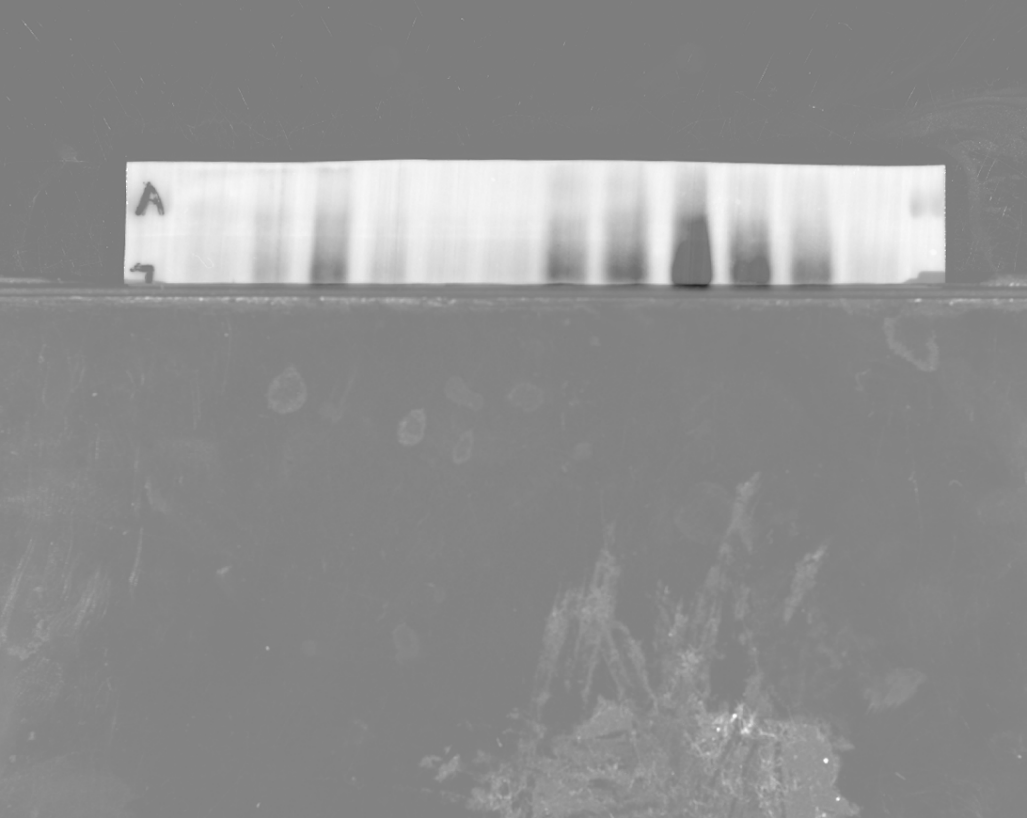


**p-mTOR**

**Atg7**

**GAPDH**

**Ctrl2**

**ESG**

**NRG**

**ESTG**

**C**


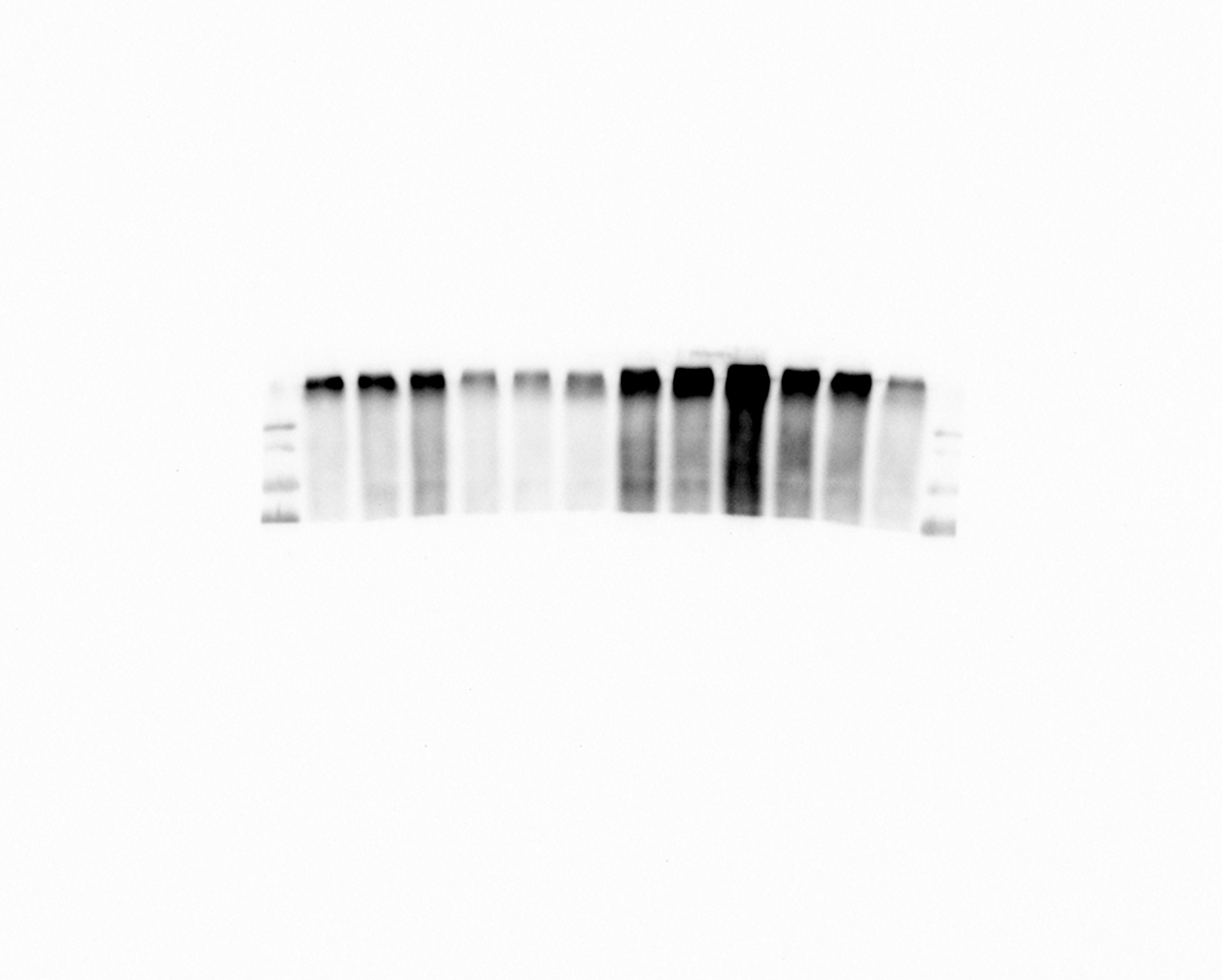


**mTOR**


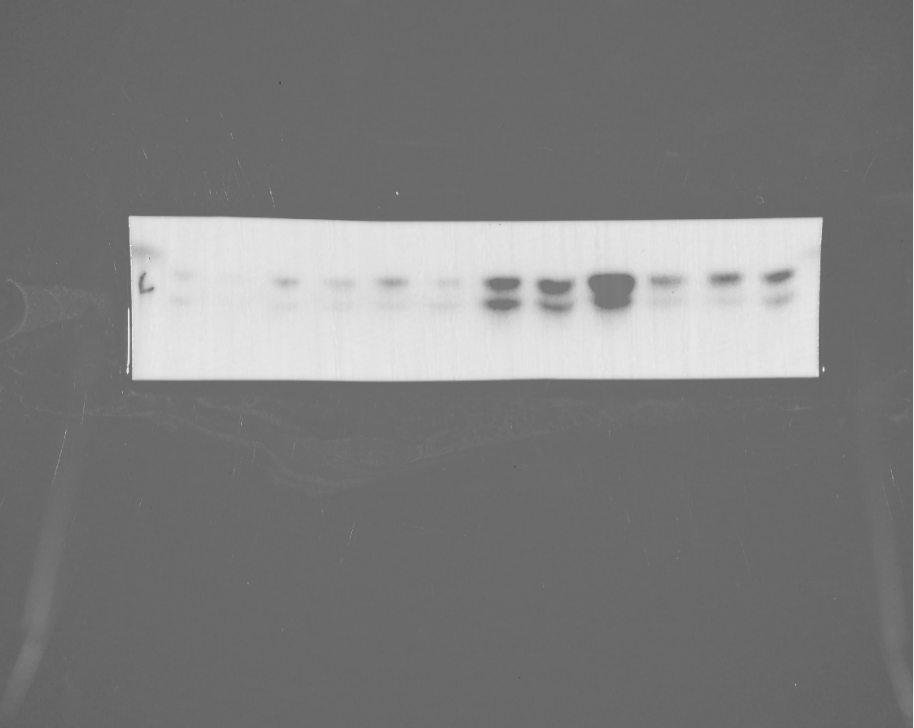


**LC3B-I**

**LC3B-II**

**Ctrl2**

**ESG**

**NRG**

**ESTG**

**17KDa**


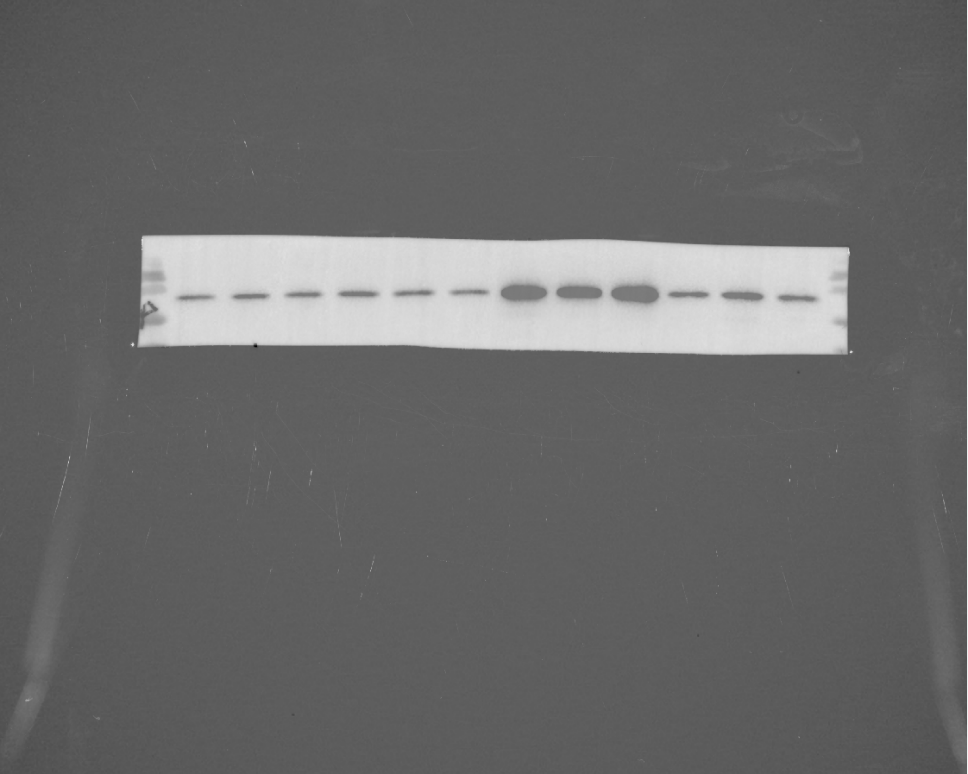


**p62**


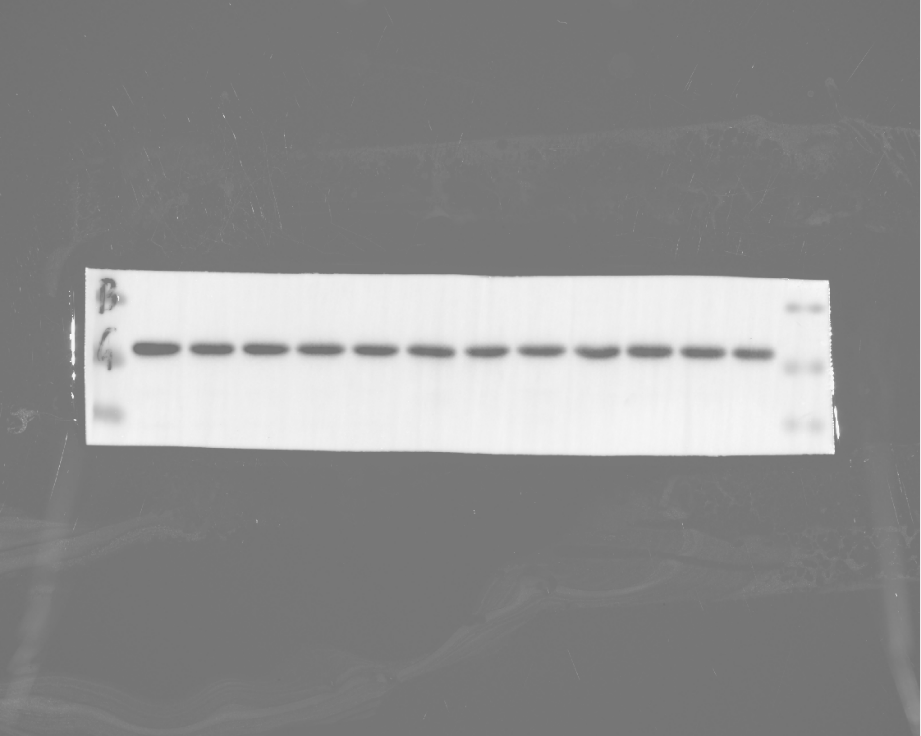


**GAPDH**

**55KDa**

**34 KDa**

**D**


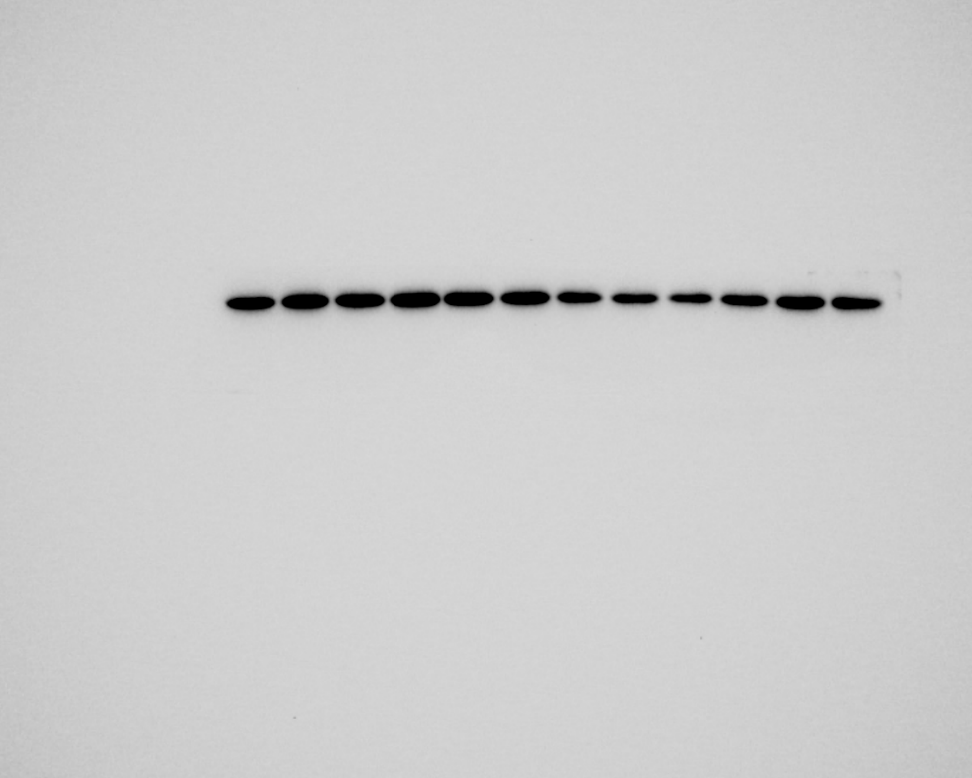


**GAPDH**
